# A novel *COL4A5* splicing variant causing X-linked Alport syndrome: A case report

**DOI:** 10.1038/s41439-022-00209-6

**Published:** 2022-08-31

**Authors:** Naonori Kumagai, Yuji Matsumoto, Tomomi Kondoh, Yohei Ikezumi

**Affiliations:** Department of Pediatrics, School of Medicine, Fujita Health University, Toyoake, Japan

**Keywords:** Alport syndrome, Disease genetics

## Abstract

Alport syndrome is a hereditary disorder characterized by renal impairment, hearing loss, and ocular symptoms and is caused by *COL4A3*, *COL4A4*, and *COL4A5* mutations. Here, we report the case of 3-year-old boy with isolated hematuria detected in routine preventative urinary screening conducted in 3-year-old children. He carried a novel variant, NM_033380.3:c. 1032 + 1 G > A, which caused a splicing abnormality in *COL4A5*. He was diagnosed with X-linked Alport syndrome.

Alport syndrome is a hereditary disorder characterized by renal impairment, hearing loss, and ocular symptoms^1^ and is caused by *COL4A3*, *COL4A4*, and *COL4A5* mutations. Historically, Alport syndrome has been diagnosed on the basis of either a combination of histopathological examination and genetic analysis or histopathological examination followed by genetic analysis. In recent years, because of advances in genetic analysis technology, the diagnosis can be made either by histopathological examination preceding genetic analysis or by genetic analysis alone^2^.

In patients with Alport syndrome, renal impairment progresses, leading to end-stage renal failure. Previously, no treatment was administered to prevent renal impairment. However, in recent years, the protective effect of angiotensin-converting enzyme (ACE) inhibitors on renal function has been reported^3,4^, and new drugs such as bardoxolone and gene therapy are under development^5,6^. Early diagnosis and treatment are crucial to improve renal function prognosis.

In Japan, routine urinary screening in 3-year-old children is useful to detect congenital anomalies of the kidneys and urinary tract. Proteinuria is considered to be a significant indicator, whereas hematuria is considered a clinically less useful indicator because serious illness is not often found in children with isolated hematuria^7^.

We report here the case of a 3-year-old child who was diagnosed with X-linked Alport syndrome by genetic analysis after hematuria was detected in routine urinary screening. This case demonstrates the usefulness of genetic analysis and urinary screening in the early diagnosis and treatment of Alport syndrome.

A 3-year-old boy was referred to Fujita Health University Hospital for further examination after isolated hematuria was identified in a routine urinary screening for 3-year-old children. His height was 93.8 cm, his weight was 12.7 kg, and his blood pressure was 70/54 mmHg. Physical examination revealed no abnormalities on chest auscultation or edema. Laboratory findings demonstrated normal renal function (estimated glomerular filtration rate, 108 ml/min/1.73 m^2^)^8^. Urinalysis showed occult blood (3+), 50–99 RBCs/high power field, and a protein/creatinine ratio of 0.16 g/gCr (Table 1). Various types of casts were observed. His mother had had hematuria with normal renal function since childhood, and his maternal grandmother had undergone hemodialysis for end-stage renal failure owing to diabetic nephropathy (Fig. 1).

**Table 1:** Laboratory findings.

Peripheral blood		Blood chemistry		Urinanalysis	
Red blood cells	4610000/μL	Glutamic-oxaloacetic transaminase	38 U/L	pH	6.5
Hemoglobin	12.2 g/dL	Glutamic-pyruvic transaminase	14 U/L	Protein	1+
Hematocrit	36.2 %	Lactate dehydrogenase	310 U/L	Occult blood	3+
Platelets	355000/μL	Alkaloine phosphatese	217 U/L	Various casts	+
White blood cells	7400/μL	Blood urea nitroge	13.6 mg/dL	Protein/Creatinine	0.16 g/gCr
		Creatinine	0.28 mg/dL	β2-MG	50> μg/L
		Cystatin C	0.81 mg/L		
		Uric acid	4 mg/dL		
		Total protein	6.6 g/dL		
		Albumin	4.3 g/dL		
		Sodium	139 mEq/L		
		Potassium	4.3 mEq/L		
		Chloride	104 mEq/L		
		Calcium	10 mg/dL		
		IP	4.9 mg/dL		
		IgG	619 mg/dL		
		IgA	94 mg/dL		
		IgM	138 mg/dL		
		C3	97 mg/dL		
		C4	16 mg/dL		
		CH50	55.5 U/mL		
		Anti-streptolysin O	10>		
		Anti-nuclear antibody	80		
		Anti-deoxyribonuckeic acid antibody	2>

**Fig. 1:**
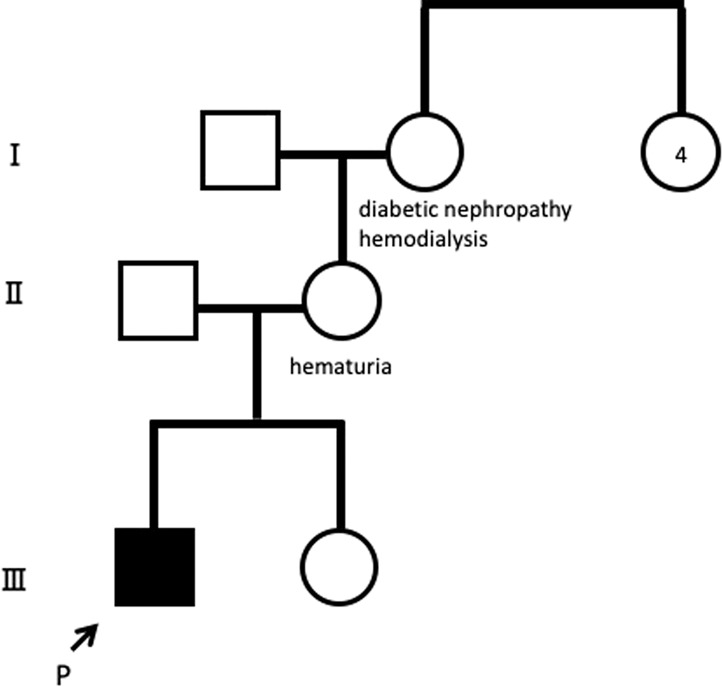
Pedigree of the family. The mother of the patient had hematuria. P proband.

After genetic counseling, we obtained consent for genetic analysis from the patient’s parents, and we performed genetic analysis of *COL4A3*, *COL4A4*, and *COL4A5* using a next-generation sequencing. The results revealed a novel hemizygous variant NM_033380.3:c. 1032 + 1 G > A, in *COL4A5*. No variants were found in the *COL4A3* or *COL4A4* genes. The patient was genetically diagnosed with X-linked Alport syndrome. No renal biopsy was performed. Treatment with an ACE inhibitor was initiated.

The hemizygous variant revealed in this patient was novel because it was not present in the Human Genetic Variation Database, Genome Aggregation Database, or ClinVar. Based on the American College of Medical Genetics and Genomics standards and guidelines, the variant was classified as pathogenic since it is categorized as a PVS1 null variant (canonical + 1 splice site)^9^ and might cause a splicing abnormality. In the present case, Alport syndrome was suspected because the patient already had marked hematuria at the age of 3 years and his mother had hematuria^10^. Although renal biopsy is preferred for the diagnosis of Alport syndrome, it is not generally indicated for isolated hematuria or hematuria with a small amount of urinary protein^10,11^. Furthermore, in Japan, the genetic analysis of Alport syndrome has been covered by public medical insurance since 2020 and can be performed in general clinical practice. Therefore, we elected to perform a genetic analysis first. The recommended timing of treatment for Alport syndrome depends on the type of inheritance and patient sex^2^. In boys with X-linked Alport syndrome, treatment is recommended after the onset of proteinuria; however, in recent years, some authors have recommended treatment from the time of diagnosis even without the onset of proteinuria^2^. Therefore, the identification of the type of inheritance is clinically useful. The patient was diagnosed with X-linked Alport syndrome before the onset of proteinuria owing to the identification of a novel variant in *COL4A5*. Genetic analysis of Alport syndrome is useful for the early diagnosis, treatment, and prognosis of Alport syndrome.

In the present case, hematuria was identified in the patient during a routine urinary screening performed in 3-year-old children, leading to the diagnosis of Alport syndrome and thus allowing early treatment initiation. In Japan, routine urinary screening in 3-year-old children is useful to identify congenital anomalies of the kidneys and urinary tract^7^. In Alport syndrome, hematuria is present from infancy^12^, and at 3 years of age, affected patients often have marked hematuria, even in the absence of proteinuria^13^. Therefore, it is possible to screen for Alport syndrome by identifying hematuria during routine urinary screening in 3-year-old children. However, because Alport syndrome was found in only 0.0032% of all urine samples from 3-year-old children^7^ and there was previously no known effective treatment, screening for Alport syndrome during routine urinalysis in 3-year-old children has never been considered. In recent years, the protective effect of ACE inhibitors on renal function has been reported. In addition, other drugs, such as bardoxolone and gene therapy, are under development^5,6^. This development indicates the recognition of the relevance of the disease and the need for an early diagnosis to improve renal prognosis. In most patients with Alport syndrome, renal function is preserved at the age of 3 years^14^; therefore, an early diagnosis of Alport syndrome based on hematuria in routine urinary screening in 3-year-old children is useful to preserve renal function. Thus, the significance of hematuria in the routine urinary screening of 3-year-old children should be reconsidered.

In conclusion, we identified a novel *COL4A5* splicing variant causing X-linked Alport syndrome in a 3-year-old child, and we demonstrated the usefulness of genetic analysis and urinary screening in a 3-year-olds for the early diagnosis and treatment of Alport syndrome.

## HGV database

The relevant data from this Data Report are hosted at the Human Genome Variation Database at 10.6084/m9.figshare.hgv.3219.
